# High-resolution anoscopy, is there a benefit in proceeding directly to the operating room?

**DOI:** 10.1007/s10151-021-02416-9

**Published:** 2021-02-10

**Authors:** B. Moeckli, J. Canner, A. Najafian, S. Carbunaru, N. Cowell, C. Atallah, E. Paredes, A. Chudnovets, S. H. Fang

**Affiliations:** 1 Department of Surgery, Ravitch Division of Colon and Rectal Surgery, High Resolution Anoscopy Clinic, The Johns Hopkins Hospital, Johns Hopkins University School of Medicine, 600 N. Wolfe St., Blalock 618, Baltimore, MD 21287 USA; 2 Department of Surgery, Geneva University Hospitals, Geneva, Switzerland; 3 Department of Plastic Surgery, Oregon Health Sciences University, Portland, OR USA; 4 Northwestern University School of Medicine, Chicago, IL USA

**Keywords:** High-resolution anoscopy, Anal cancer, High-grade dysplasia, Anal pap smear, Patient comfort

## Abstract

**Background:**

The development of high-resolution anoscopy (HRA) has advanced our ability to detect anal dysplasia. Historically, HRA is performed in a clinical setting and subsequent ablation is performed in the clinical setting or operating room. The aim of this study was to determine the most effective venue for the performance of HRA.

**Methods:**

Following institutional review board (IRB) approval, the correlation between anal cytology and HRA performed in the clinic versus in the operating room was evaluated. Data were extracted from our IRB-approved prospective HRA database over the time period of 2013–2017.

**Results:**

One hundred twenty-eight HRAs were compared (101 in the clinical setting, 27 in the operating room). There was a statistically significant difference in the correlation between anal cytology and HRA pathology for procedures performed in the clinical setting (55% [56/101]) versus those performed in the operating room (82% [22/27]) (*p* = 0.014). More biopsies were obtained in the operating room than in the clinic setting (3 vs. 1, *p* < 0.0001). The majority of patients who had HRA in a clinical setting with subsequent HRA in the operating room stated that they preferred to have their HRAs performed in the operating room due to discomfort from the HRA procedure.

**Conclusions:**

Detection rates for anal dysplasia on HRA, are significantly higher when performed in the operating room. To prevent discomfort in the clinical setting, patients with high-grade dysplasia on anal pap testing may benefit from proceeding directly to the operating room for concurrent HRA and ablation.

## Introduction

Each year 8200 new cases of anal cancer are responsible for close to 1100 deaths in the United States [1]. Although anal squamous neoplasms only represent a small proportion of the total number of cancer cases of digestive tract neoplasms, its incidence has risen over the past few decades while survival has not improved [2, 3]. In addition, certain subgroups of patients, which include human immunodeficiency virus (HIV)-positive men who have sex with men (MSM), have a significantly increased risk of developing anal cancer. The yearly incidence of anal cancer for HIV-positive MSM in the United Stated is reported to be 137 per 100,000 compared to 1.5 per 100,000 for the general population [4], a rate that exceeds the incidence of cervical cancer prior to the introduction of a widespread screening program in England [5].

A number of similarities exist between cervical cancer and anal squamous cell carcinoma (ASCC). Both occur at a squamocolumnar junction, a transitional zone characterized by high cell turnover, and are associated with high-risk human papillomavirus (HPV) infection [6]. Furthermore, both exhibit a stepwise progression from precancerous lesions to invasive carcinoma [7]. HPV-associated progression ranges from normal squamous cell epithelium to low-grade squamous intraepithelial lesions (LSIL) to high-grade squamous intraepithelial lesions (HSIL), and ultimately to invasive cancer [8, 9], with a median transformation time from high-grade dysplasia to invasive neoplasia ranging from 14 to 120 months [10, 11]. Patients with early anal cancer have an excellent 5-year survival rate of > 80%. Whereas those with advanced disease with distant metastasis have a 5-year survival rate of 30% [12].This highlights the importance of early detection.

One of the major public heath success stories of the past century has been the widespread introduction of cytology based Papanicolaou (Pap) test to detect pre-neoplastic lesions in cervical cancer, resulting in a drastic reduction in mortality [13]. Analogously anal cancer lends itself to a targeted screening program. This is due to a clearly defined high-risk group, combined with a long latent phase and markedly improved survival for early versus late stage disease. Professional society guidelines from the American Society of Colon and Rectal Surgeons (ASCRS) recommend screening of high-risk individuals with anal Pap tests [2]. Suspicious lesions from the anal Pap test are further characterized with high-resolution anoscopy (HRA).

Historically, HRA is performed in a clinical setting whereas subsequent ablation is accomplished either in a clinic-based setting or in the operating room (OR). However, to our knowledge there are no objective data, which indicate which setting is best for performing HRA. The aim of this study was to determine the best setting for the performance of HRA.

## Materials and methods

### Study design

Data were obtained from our prospectively maintained institutional review board (IRB)-approved HRA database. All patients, aged 18 years and older who had HRA with biopsy, between November 1, 2011 and July 30, 2017, were reviewed from our IRB-approved prospective HRA database. Patients who did not provide written informed consent to be a part of the HRA database, had HRA without biopsies, or did not have biopsy results available, were excluded. Repeat HRAs were also excluded to avoid confounding since HRAs in the OR were more frequently repeat procedures.

### Anal cytology and HRA

Anal Pap tests and HRAs were performed in our weekly HRA clinic. Patients were referred to the clinic from their primary care physicians or our institution’s HIV clinic if they had a history of abnormal Pap test results.

In clinic, patients were placed in the left lateral decubitus position with their knees tucked to their chest. For anal cytology, a Dacron swab was inserted into the anus with the tip just proximal to the dentate line. The swab was rotated within the anal canal for at least 30 s, while withdrawing the swab, to obtain a proper anal Pap specimen. A baseline anoscopy was performed to visualize the anal canal. Five-percent acetic acid soaked gauze was placed at the dentate line for 2 min and then removed. Under colposcopic visualization, the dentate line was identified and neovascular acetowhite lesions were identified and biopsied with an endoscopic cold biopsy forceps. Lugol’s solution was applied and inspected with the colposcope. Suspicious Lugol’s negative lesions were biopsied with an endoscopic cold biopsy forceps. Monsel’s solution was applied for hemostasis. Anal cytology and HRA biopsies were reviewed by the Pathology Department at the Johns Hopkins Hospital.

In the operating room, the patient was placed in prone jack-knife position, under either conscious sedation or general anesthesia, with administration of a local perianal field block.

Cytological findings were categorized as no dysplasia, atypical squamous cells of undetermined significance (ASCUS), low-grade dysplasia (LSIL), or high-grade dysplasia (HSIL, low-grade squamous intraepithelial lesions suspicious for HSIL, and atypical squamous cells suspicious for HSIL).

Histopathological results from the anal biopsies obtained during the HRA were classified using the most recent two-tiered classification system recommended by the Lower Anogential Squamous Terminology Project [8]. The most severe biopsy result from each patient was used if several biopsies were obtained. Condyloma and Anal Intraepithelial Neoplasia (AIN)—1 were classified as LSIL, AIN-2 and AIN-3 were categorized as HSIL.

We determined that there was a correlation between the HRA and anal cytology in the following scenarios:

- If anal cytology and the histopathological assessment of the anal biopsy were in the same category according to the two-tiered classification system (HSIL, LSIL or no dysplasia); i.e., HSIL on anal cytology matched HSIL on anal biopsy and so forth.
- If anal cytology was ASCUS and the histopathological assessment of the anal biopsy was HSIL, LSIL, or no dysplasia
  We determined that there was no correlation between HRA and anal cytology in the following scenarios:
- If anal cytology was HSIL but the biopsies obtained during the HRA were low grade or negative
- If anal cytology was LSIL and the biopsies obtained during HRA were HSIL.

### Statistical analysis

Independent t-tests and the Kruskal wallis test were used to compare groups with respect to continuous variables. All variables were presented as medians with interquartile ranges (IQR). Counts and proportions were analyzed using chi-square tests. All statistical analyses were performed using Stata®/MP 14.2 (StataCorp LLC, College Station, TX, USA). A *p* value of < 0.05 was considered to be significant.

## Results

Overall 128 patients were included in this retrospective study, of which 27 underwent HRA in in the OR and 101 in a clinic setting. A summary of patient demographic data is presented in Table 1 (Table 1). The proportion of men in the group that underwent HRA in the OR was higher. A higher percentage of patients who had HRA in the OR are current smokers, although this did not reach statistical significance. There was no statistically significant difference in age, body mass index, HIV status, comorbidity, or race between the two study groups. The number of biopsies obtained per procedure was significantly higher in the OR group with a median of 3 (range 2–4) biopsies compared to 1 (range 1–2) biopsy for the clinic group (*p* =  < 0.0001). There was a trend toward more post procedural bleeding and pain in the OR group which did not reach statistical significance (*p* = 0.16 and *p* = 0.64, respectively) (Table 2).

**Table 1:** Demographic and clinical characteristics of patients having high-resolution anoscopy

Characteristic		OR (*n* = 27)	Clinic (*n* = 101)	*p* value
Age (years), median (IQR)		47 (41–54)	49 (39–54)	0.72
Sex: male		14 (52%)	31 (31%)	0.04
BMI (kg/m^2^), median (IQR)		29.0 (22.0–31.5)	26.6 (23.6–28.4)	0.08
Race	Asian	0 (0%)	2 (2%)	
	Caucasian	12 (44%)	46 (47%)	
	Black	13 (48%)	44 (45%)	0.36
	Hispanic	1 (4%)	1 (1%)	
	Other	0 (0%)	3 (3%)	
HIV +		18 (67%)	74 (73%)	0.50
CD4 count < 200^b^		1 (6%)	9 (12%)	0.46
Sexual preference	MSM	10 (37%)	38 (40%)	
	Heterosexual	7 (26%)	17 (18%)	0.19
	Unknown ^c^	9 (33%)	41 (43%)	
Smoking (current)		20 (74%)	52 (51%)	0.09
Comorbidities	Hypertension	7 (26%)	22 (22%)	0.65
	Diabetes	1 (4%)	6 (6%)	0.64
	Renal insufficiency	2 (7%)	3 (3%)	0.29
	Coronary artery disease	0 (0%)	4 (4%)	0.29
	Ulcerative colitis	1 (4%)	1 (1%)	0.51

*BMI* body mass index, *MSM* men who have sex with men, *HIV* human immunodeficiency virus

^a^Data are presented as number (percentage) unless otherwise noted

^b^The CD4 count was only available for 91 of the patients and the *p* value was calculated based on the available data

^c^Includes missing data and data of patients who declined disclosure of their sexual orientation

**Table 2:** Comparison of number of biopsies and complications between HRA performed in the OR and clinic

	OR (*n* = 27)	Clinic (*n* = 101)	*p* value
Number of biopsies, median (IQR)	3 (2–4)	1 (1–2)	< 0.0001
Post procedural bleeding *n* (%)	1 (4)	1 (1)	0.16
Post procedural pain *n* (%)	5 (19)	6 (6)	0.64

*HRA* high-resolution anoscopy, *OR* operating room

**Fig. 1:**
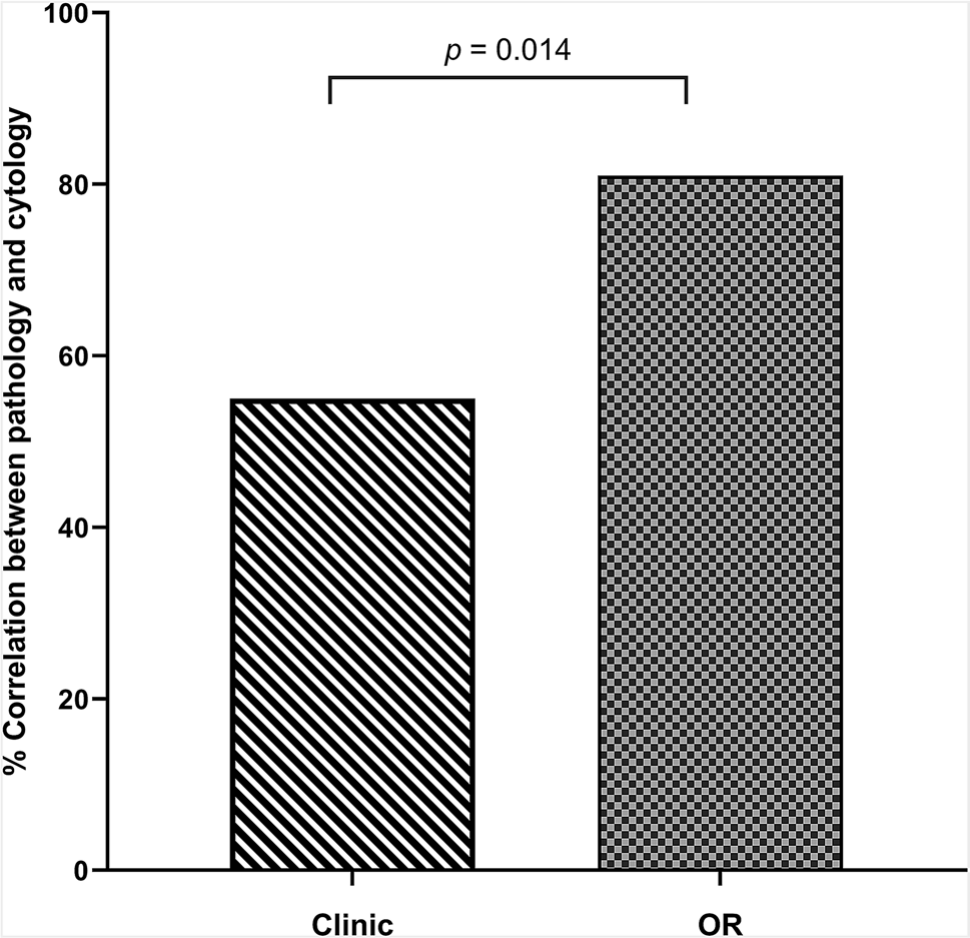
Correlation between pathology and cytology from high-resolution anoscopy performed in clinic compared to the operating room. *OR* Operating room, Pearson *χ*^2^(1) = 6.0670, *p* value = 0.014

We observed an increased correlation between anal cytology and HRA pathology in the OR group that was statistically significant with a concordance of 81% compared to a correlation of 55% in the clinic group (*p* = 0.014) (Fig. 1).

## Discussion

HRA is a technique that was first described in 1989 by Scholefield et al. [14] and first performed in the United States by Dr. Michael Berry at University of California-San Francisco. [15]. The benefits of HRA are uncontested and its main utility lies in increased sensitivity and specificity for detecting pre-cancerous lesions compared to cytology-based methods [16, 17]. This is further underlined by a Canadian study that identified the use of HRA as the most cost-effective screening strategy relative to other screening modalities including anal Pap tests for the detection of high-grade anal intraepithelial neoplasia [18]. Yet anal cytology and HRA can be used as complementary tests. A finding of high-grade dysplasia on an anal pap test can be indicative of the presence of high-grade pathology on HRA [16, 19].

HRAs performed in the OR exhibit a significantly increased correlation to anal cytology with an 82% correlation for procedures performed in the OR versus only 51% correlation to anal cytology for HRA procedures performed in clinic. This increased correlation suggests a higher quality exam when the procedure is performed in the OR. A high discordance of cytology and histology can indicate that a dysplastic lesion has been missed on HRA. In cervical cancer screening practice, cytology and colposcopic-directed biopsy pathology correlation has been used as a quality measure [20] and prior studies show that the majority of discrepancies between colposcopic-directed biopsy pathology and cytology can be explained by sampling error during colposcopic-directed biopsy [21, 22]. Given the striking similarities between anal and cervical cancer screening, we believe that the same situation holds true for anal cytology and HRA biopsy correlation and that sampling errors could be markedly reduced by performing HRAs in the OR.

A recently published prospective study by Roberts et al., shows a clear improvement of the positive predictive value (PPV) of detecting histological HSIL on HRA when the baseline cytology is HSIL over a 12-month period, increasing from 72 to 93%. This finding suggests that foci of HSIL are missed on the baseline HRA examination and an examination in the OR can potentially help to reduce the number of HRA procedures necessary to accurately diagnose pre-neoplastic lesions.

There are several reasons why performing HRA in the OR may increase detection rate of anal dysplasia. In the OR, patients are subjected to anesthesia and the surgeon is able to place larger anal retractors for better visualization of abnormal lesions. In addition, the anorectal examination is not rushed due to patient discomfort. The patient is placed in prone jack-knife position so that anorectal anatomy is better visualized.

One factor that may have contributed to the higher correlation of anal cytology and HRA pathology is the significantly higher number of biopsies obtained in the OR versus that obtained in clinic. With a median number of three biopsies in the OR, as opposed to one, when performed in clinic, the probability of picking up a dysplastic lesion increases. This is supported by the work of Silvera et al., [23] which showed that taking random biopsies of normal appearing quadrants increased the number of diagnoses of HSIL identified per patient. However, this study reported only an increase of patients with HSIL detected of 9.8% and therefore would not account for the entirety of the effect that we observed in this study.

Although there are currently no randomized controlled trials looking at the effectiveness of treatment of HSIL several retrospective studies have shown that treatment of high-grade anal dysplasia is both effective in preventing anal cancer and cost effective [24–26].

Previously we performed all screening HRA in our outpatient clinic. After the analysis and comparison of the results between HRAs obtained in the OR and in the outpatient clinic we have revised our patient pathways. We currently perform a concurrent anal Pap test and HRA in clinic. If the anal pap test results in high-grade pathology (HSIL), our protocol is to proceed directly to the operating room for HRA with biopsy and ablation, due to our findings that visualization of high-grade lesions in the operating room is superior to visualization in a clinic setting. In addition, if there is any concern about having an awake procedure in clinic or the patient is uncomfortable during the initial examination, then the surgeon will offer to complete the HRA in the operating room. This strategy has several advantages it eliminates the need for an additional procedure and avoids the discomfort associated with an office based HRA. This strategy may also increase patient’s compliance to follow-up. Of the seven procedures that were aborted in our cohort, all of them were performed in the outpatient clinic and five out of seven of the cases were terminated due to patient discomfort.

The limitations of our study also deserve discussion. Our sample size was relatively low. In addition, there is the issue of biased patient selection; that is, patients that were seen in HRA clinic had already been pre-screened as “high-risk” because the majority were referred by our institution’s HIV care center. As such, our findings are not applicable to large populations, but should be considered a preliminary description of risk that could potentially be applied in appropriate settings such as HIV clinics. Finally, while patients were more comfortable and there was better visualization of the anal canal when the HRA was performed in the operating room, the patients were subjected to the risks of anesthesia.

## Conclusions

Our results suggest that proceeding directly to the OR for HRA in the appropriately selected, high-risk patients with HSIL on anal Pap test is beneficial. It could result in a higher detection rate of dysplastic lesions, increase patient comfort during the procedure and ablation, and decrease the number of unnecessary procedures.
